# Measles Immune Suppression: Lessons from the Macaque Model

**DOI:** 10.1371/journal.ppat.1002885

**Published:** 2012-08-30

**Authors:** Rory D. de Vries, Stephen McQuaid, Geert van Amerongen, Selma Yüksel, R. Joyce Verburgh, Albert D. M. E. Osterhaus, W. Paul Duprex, Rik L. de Swart

**Affiliations:** 1 Viroscience Lab, Erasmus MC, Rotterdam, The Netherlands; 2 Tissue Pathology, Belfast Health and Social Care Trust, Queen's University of Belfast, Belfast, Northern Ireland, United Kingdom; 3 Department of Microbiology, Boston University School of Medicine, Boston, Massachusetts, United States of America; Duke-NUS Graduate Medical School, Singapore

## Abstract

Measles remains a significant childhood disease, and is associated with a transient immune suppression. Paradoxically, measles virus (MV) infection also induces robust MV-specific immune responses. Current hypotheses for the mechanism underlying measles immune suppression focus on functional impairment of lymphocytes or antigen-presenting cells, caused by infection with or exposure to MV. We have generated stable recombinant MVs that express enhanced green fluorescent protein, and remain virulent in non-human primates. By performing a comprehensive study of virological, immunological, hematological and histopathological observations made in animals euthanized at different time points after MV infection, we developed a model explaining measles immune suppression which fits with the “measles paradox”. Here we show that MV preferentially infects CD45RA^−^ memory T-lymphocytes and follicular B-lymphocytes, resulting in high infection levels in these populations. After the peak of viremia MV-infected lymphocytes were cleared within days, followed by immune activation and lymph node enlargement. During this period tuberculin-specific T-lymphocyte responses disappeared, whilst strong MV-specific T-lymphocyte responses emerged. Histopathological analysis of lymphoid tissues showed lymphocyte depletion in the B- and T-cell areas in the absence of apoptotic cells, paralleled by infiltration of T-lymphocytes into B-cell follicles and reappearance of proliferating cells. Our findings indicate an immune-mediated clearance of MV-infected CD45RA^−^ memory T-lymphocytes and follicular B-lymphocytes, which causes temporary immunological amnesia. The rapid oligoclonal expansion of MV-specific lymphocytes and bystander cells masks this depletion, explaining the short duration of measles lymphopenia yet long duration of immune suppression.

## Author Summary

Measles is associated with a transient immune suppression, resulting in increased susceptibility to opportunistic infections. Indeed, the main causes of measles mortality are secondary infections in the respiratory and digestive tract. Although measles is associated with lymphopenia, depletion of lymphocytes has often been dismissed as a cause of immune suppression. Lymphocyte counts rapidly return to normal after clearance of the virus, while immune suppression lasts several weeks to months. Many studies have focused on suppression of lymphocyte proliferation as an *in vitro* correlate of immune suppression. However, experimental infections of non-human primates show that *in vivo* lymphocyte proliferation is not impaired after measles. Instead, we hypothesize that massive expansion of MV-specific and bystander lymphocytes masks the fact that pre-existing memory lymphocytes have been depleted. We conclude that measles virus infection wipes out immunological memory, leaving individuals susceptible to opportunistic infectious agents that would normally be controlled by the immune system.

## Introduction

Measles is associated with a transient but profound immune suppression, which may last for several weeks to months after the acute stage of the disease. The clinical importance of this immune suppression is illustrated by the observation that measles mortality is typically caused by secondary infections in the respiratory or digestive tract [1]–[3]. However, the mechanism by which measles virus (MV) infection causes immune suppression is not completely understood. Multiple *in vivo* correlates of immune suppression have been described, including disappearance of Mantoux responses [4], [5], lymphopenia [6], [7] and impaired responses to vaccination [8], [9]. Decreased lymphoproliferative responses [10], [11], altered cytokine response profiles [12] and impairment of antigen-presenting cell function [13]–[15] have been described *in vitro*. The relevance of these observations to immune suppression and enhanced susceptibility to opportunistic infections remains unclear. The paradox of measles is that the acute phase of the disease is not only associated with immune suppression, but also with immune activation [16] and induction of robust MV-specific humoral and cellular immune responses that result in lifelong immunity.

MV infection is initiated in the respiratory tract. It has long been thought that the initial target cells of the virus were epithelial cells of the upper respiratory tract, but recent studies have demonstrated a major role for alveolar macrophages and dendritic cells (DC) in this process [17], [18]. These first MV-infected cells transmit the virus to the bronchus-associated lymphoid tissue (BALT) and/or the draining lymph nodes, where the infection is further amplified in lymphocytes and viremia is initiated [18], [19]. MV infects both T- and B-lymphocytes by binding of the MV-hemagglutinin (H) glycoprotein to the cellular receptor CD150 [20]. Recently, the adherens junction protein PVRL4 was identified as cellular receptor on epithelial cells [21], [22]. However, as this receptor is exclusively expressed on the basolateral surface of epithelial cells, it does not facilitate MV infection of epithelial cells during the early stages of the disease, but instead is thought to play a role in transmission during the late stages of measles pathogenesis [23].

It has been proposed that direct infection of lymphocytes and the subsequent lymphopenia could explain measles-associated immune suppression [24], [25]. However, this hypothesis has often been dismissed based on the observation that lymphopenia only lasts about a week, whilst immune suppression persists for several weeks to months. In addition, during the peak of viremia no more than 1 to 5% of the total lymphocyte population in peripheral blood is infected [26], [27]. Recent observations that MV infects high percentages of cells in lymphoid tissues [27] and preferentially targets CD45RA^−^ or CD45R0^+^ memory T-lymphocytes [27], [28] led us to revisit the lymphocyte depletion hypothesis for immune suppression using the macaque model. Analysis of virological, immunological and histopathological parameters has demonstrated a remarkable similarity between measles in macaques and humans [29]. Here we present a comprehensive overview of a number of *in vivo* studies performed in macaques which provides a unifying model for the etiology of measles immune suppression that is both compatible with the measles paradox and with historical *in vitro* and *in vivo* correlates of measles immune suppression.

## Results

### MV targets lymphoid tissues and preferentially infects CD45RA^−^ memory T-lymphocytes

**Figure 1:**
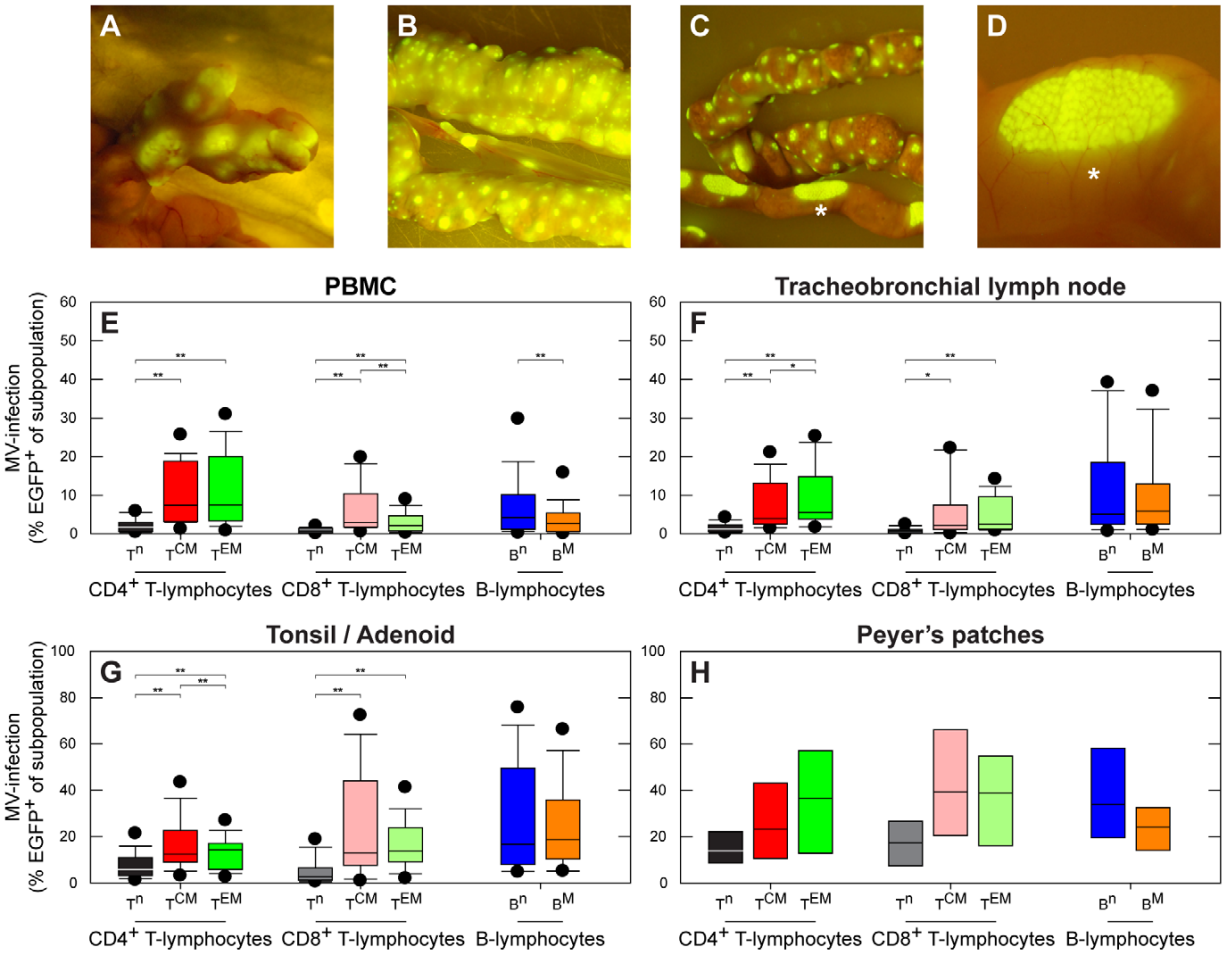
MV infects high percentages of B-lymphocytes and CD45RA^−^ memory T-lymphocytes. (A–D) Macroscopic detection of EGFP in lymphoid tissues of the gastro-intestinal tract in three different macaques: mesenteric lymph nodes (A), gut-associated lymphoid tissue (GALT) (B and C), including the Peyer's patches (C and D). Panel D is an enlargement of panel C (indicated by asterisk); (E–H) MV infection percentages in lymphocyte subsets during the approximate peak viremia. T-lymphocyte subpopulations were identified as T^n^ (CD45RA^+^), T^CM^ (CD45RA^−^CCR7^+^) or T^EM^ (CD45RA^−^CCR7^−^), B-lymphocytes were identified as B^n^ (CD27^−^IgD^+^) or B^M^ (CD27^+^IgD^−^). Box plots were chosen since the data were not normally distributed, and show the median infection percentages with the 25^th^–75^th^ percentiles, error bars indicate the 10^th^–90^th^ percentiles, dots the 5^th^–95^th^ percentiles. The 10^th^–90^th^ percentiles and 5^th^–95^th^ percentiles are only shown if the number of observations is at least ten; **, *P*<0.01. *, *P*<0.05. In panel E, F and G 14 animals were included; in panel H 3 animals were included.

We have analyzed data from rhesus or cynomolgus macaques (n = 40) infected with recombinant (r) MV strains (rMV^IC323^ or rMV^KS^) expressing enhanced green fluorescent protein (EGFP), spanning the early, intermediate and late stages of MV infection (Table S1). Data from previous studies [18], [27], [30] and from additional experimentally infected macaques (n = 14) were combined. The rMVs expressed EGFP from an additional transcription unit, and MV replication results in the host cell becoming EGFP^+^. At the peak of viremia, EGFP fluorescence was macroscopically detected in all lymphoid tissues (Figure 1A–D). The percentages MV-infected cells in lymphocyte subsets in PBMC or lymphoid tissues collected 9 or 11 days post-infection (d.p.i.) were determined by flow cytometry. Lymphocytes were subtyped as CD4^+^ or CD8^+^ naive (CD45RA^+^, T^n^), central memory (CD45RA^−^CCR7^+^, T^CM^) or effector memory (CD45RA^−^CCR7^−^, T^EM^) T-lymphocytes or as naive (IgD^+^CD27^−^, B^n^) or memory (IgD^−^CD27^+^, B^M^) CD20^+^HLA-DR^+^ B-lymphocytes (Figure S1). T^CM^ and T^EM^ were infected at a significantly higher level than T^n^. In contrast, B^M^ were not preferentially infected (Figure 1E–H).

**Figure 2:**
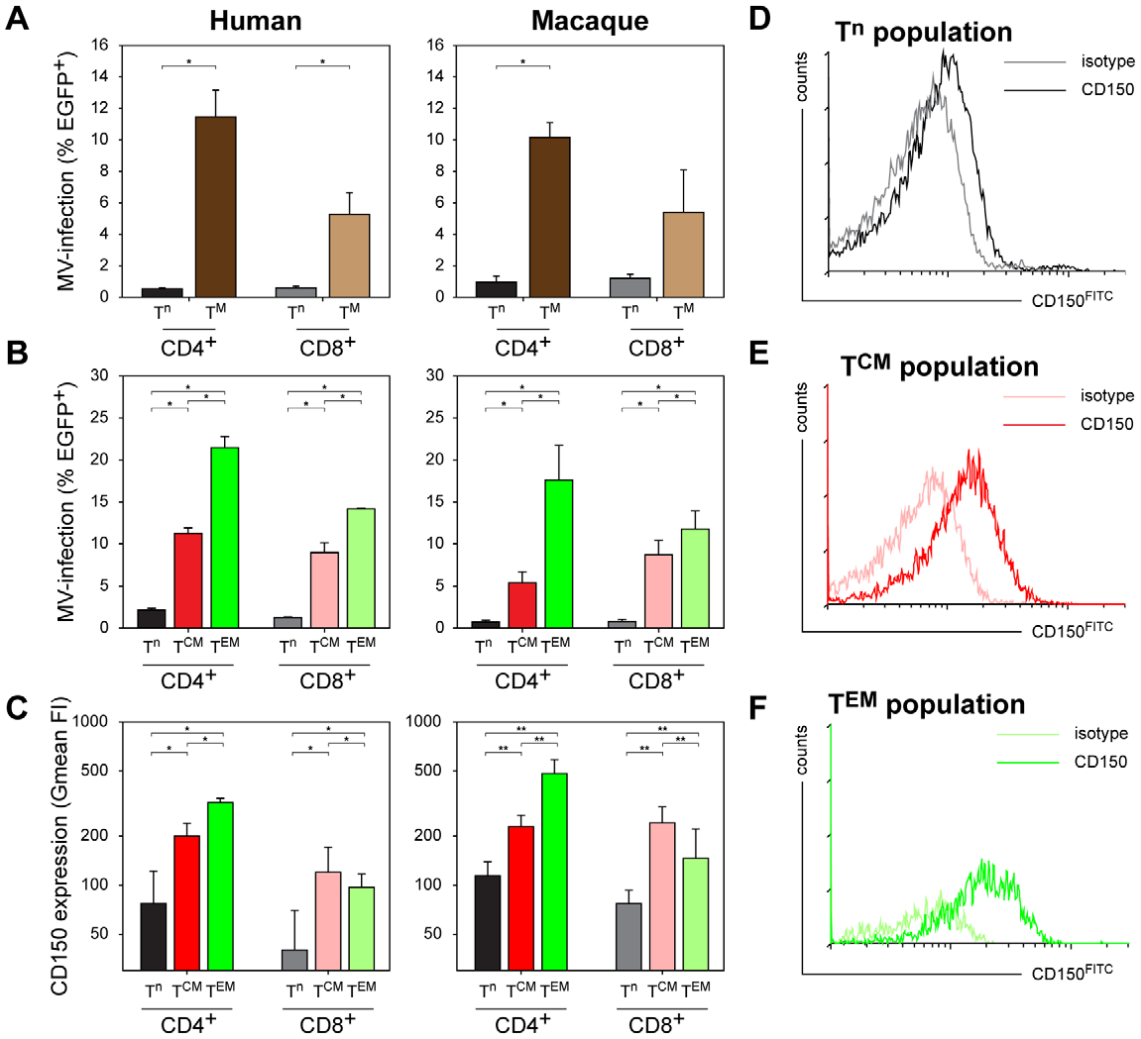
Susceptibility of human or macaque T-lymphocyte subsets to *in vitro* MV infection. (A) Human or macaque PBMC were sorted into naive (CD45RA^+^, T^n^) or memory (CD45RA^−^, T^M^) CD4^+^ or CD8^+^ T-lymphocytes, and infected with MV *in vitro*. Percentages MV-infected T-lymphocytes were determined 2 d.p.i. by measuring EGFP fluorescence by flow cytometry. CD4^+^ (human and macaque) and CD8^+^ (human only) T^M^ were significantly more susceptible to MV infection than the corresponding T^n^. For macaque CD8^+^ T-lymphocytes the difference was significant in two out of three experiments; (B) Unsorted human and macaque PBMC were infected, MV infection percentages in the different T-lymphocyte subsets were determined 2 d.p.i. by flow cytometry. Both in human and macaque PBMC the CD4^+^ and CD8^+^ T^CM^ and T^EM^ were significantly more susceptible to MV infection than the corresponding T^n^ subpopulations. In addition, CD4^+^ T^EM^ and, to a lesser extent, CD8^+^ T^EM^ proved more susceptible to MV infection than T^CM^. (C–F) Levels of CD150 expression on the different T-lymphocyte subsets in human and macaque PBMC. (C) PBMC collected from human or macaque donors were stained for memory markers as described in Figure S1, in combination with an IgG1 isotype control or CD150^FITC^ staining. CD150 expression on the different subsets is shown as geometric mean fluorescence intensity (Gmean FI) ± SD. Both for humans and macaques CD150 expression on CD4^+^ and CD8^+^ T^CM^ and T^EM^ was significantly higher than on T^n^. Interestingly, in CD4^+^ T-lymphocytes CD150 expression was significantly higher on T^EM^ than on T^CM^, whereas in CD8^+^ T-lymphocytes an inverse pattern was observed. (D–F) An IgG1 isotype control was used to determine the level of background staining, and is shown in combination with the CD150 staining for each subset. **, *P*<0.01. *, *P*<0.05. Experiments were performed with sorted cells from 3 macaque and 2 human donors, and unsorted cells from 4 macaque and 5 human donors. Data are shown as means ± standard deviation (SD) of representative donors.

To determine whether the increased susceptibility of CD45RA^−^ memory T-lymphocytes (T^M^) to MV infection is an inherent property of these cells, and comparable between humans and macaques, we sorted naive and memory CD4^+^ and CD8^+^ T-lymphocyte populations from human or macaque PBMC on basis of CD45A expression. *In vitro* co-culture of these populations with MV-infected autologous cells showed that both human and macaque CD45RA^−^ T^M^ were preferentially infected by MV (Figure 2A). In an alternative approach, unsorted human or macaque PBMC were co-cultured with autologous MV-infected cells, and infection percentages in T^n^, T^CM^ and T^EM^ were determined by flow cytometry, resulting in similar differences in the susceptibility of T-lymphocyte subpopulations (Figure 2B). This not only corroborated the *in vivo* results from the experimentally infected macaques, but also demonstrated a virtually identical trend for human and macaque subpopulations.

The MV receptor CD150 is expressed at high levels by activated human CD45RO^high^ memory T-lymphocytes [31]. We determined the expression levels of CD150 on the different human and macaque T-lymphocyte subsets. This confirmed the higher level of CD150 expression by human CD45RA^−^ T^CM^ and T^EM^ when compared to CD45RA^+^ T^n^, and showed that the expression levels of CD150 on human and macaque T-lymphocyte subsets are comparable, which likely explains the increased susceptibility of CD45RA^−^ T^M^ to MV infection (Figure 2C–F). Although we were unsuccessful in further subtyping B-lymphocyte subsets on basis of expression patterns of different surface markers, the observed efficient infection of both B^n^ and B^M^ lymphocytes seems in accordance with recent studies demonstrating CD150 expression on virtually all human B-lymphocyte subpopulations [32].

### MV causes lymphocyte depletion in lymphoid tissues

**Figure 3:**
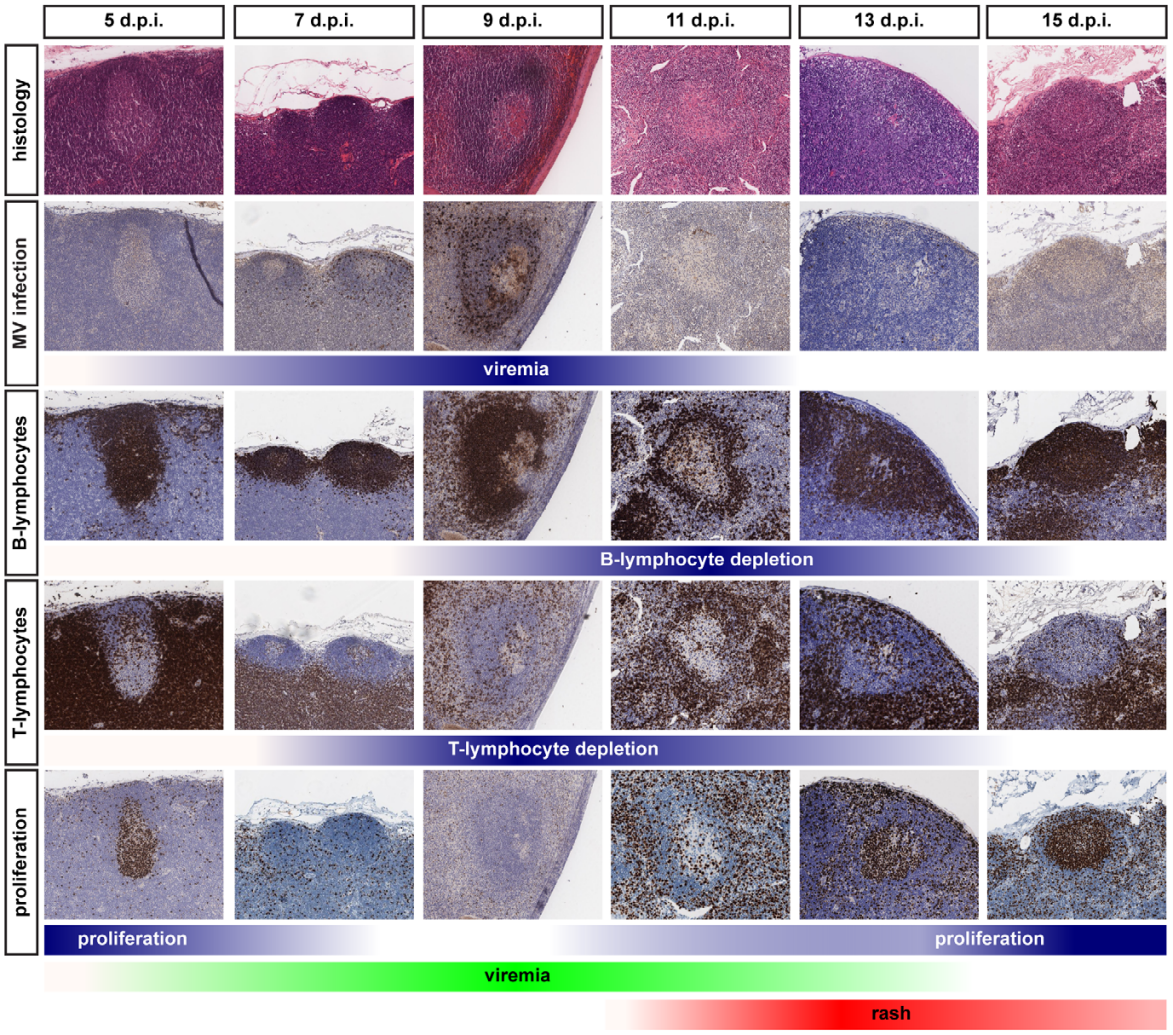
Histology and immunohistochemistry of lymphoid tissues obtained from macaques euthanized between 5 and 15 d.p.i. Serial sections were stained for histological changes (H&E), MV infection (EGFP), B-lymphocytes (CD20), T-lymphocytes (CD3) or proliferating cells (Ki67). Multiple lymphoid tissues from multiple animals collected at each time-point were analyzed, panels shown are representative for the tissues that have been examined. The color intensity of the blue bars below the photomicrographs indicates the relative levels of viremia, lymphocyte depletion or proliferation. The green and red bars at the bottom indicate the appearance and disappearance of viremia and rash and correspond to the bars in Figure 5B.

To address the impact of MV infection *in situ*, immunohistochemical analyses of serial sections of lymphoid tissues collected at different d.p.i. were performed. This demonstrated that MV mainly replicated in B-cell follicles (Figure 3; 7 and 9 d.p.i.), as previously described [27], [33]. Multiple syncytia were observed 9 d.p.i., and dual immunofluorescence showed these were of B-lymphocyte origin (Figure S2). Strikingly similar to classic observations in humans [34], lymphoid exhaustion of the centers of the B-cell follicles was observed during and shortly after the peak of viremia (Figure 3; 9 and 11 d.p.i.).

### MV infection suppresses tuberculin-specific T-lymphocyte responses

**Figure 4:**
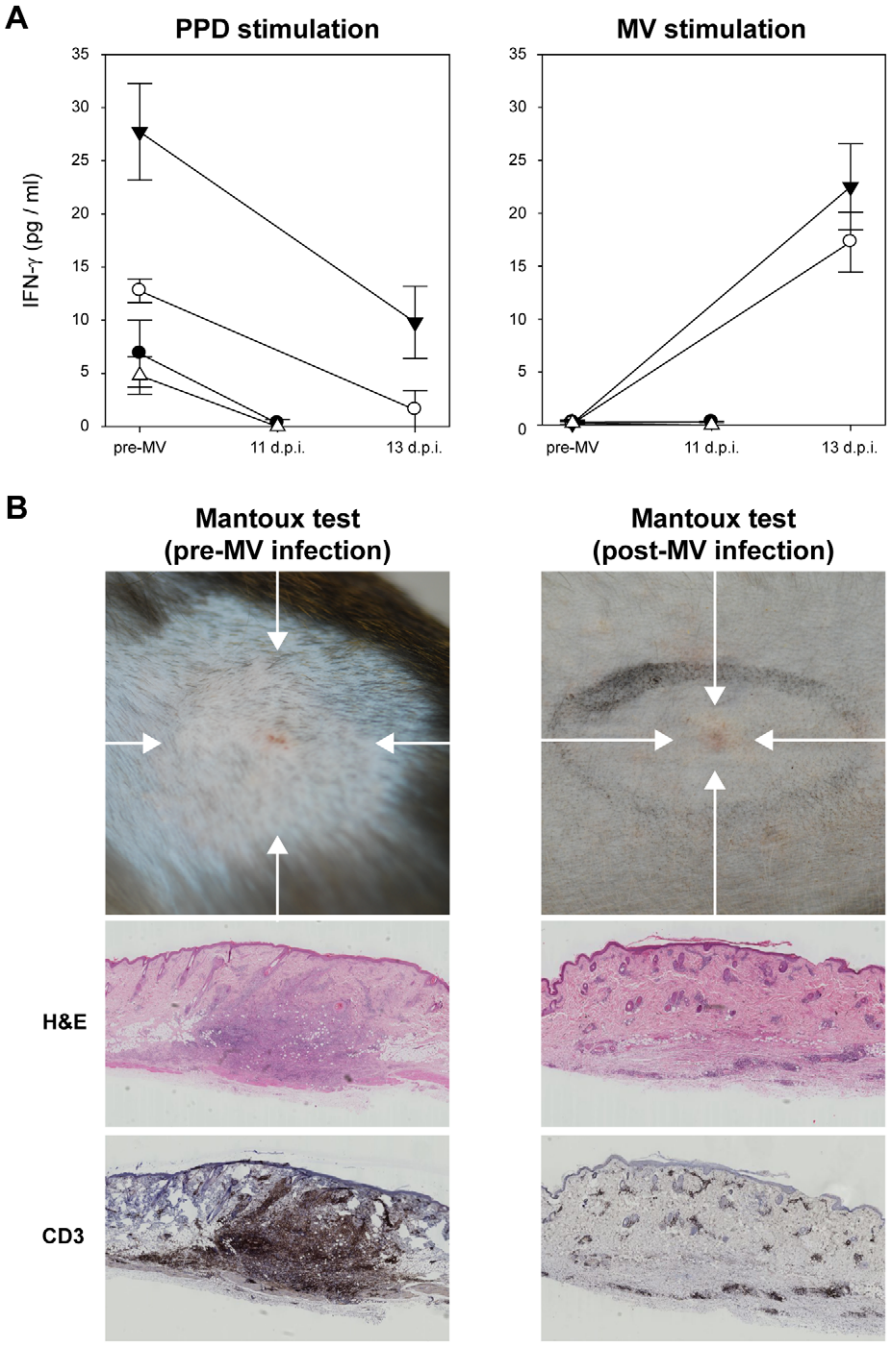
MV infection causes temporary immunological amnesia. (A) T-lymphocyte responses to PPD and MV were measured by IFN-γ production after *in vitro* stimulation of PBMC collected from 4 BCG-vaccinated macaques. Measurements were performed in triplicate, graphs shows means ± SD; (B) Mantoux tests were performed 7 days before (n = 4) and 8 (n = 2) or 10 (n = 2) days after MV infection. Images were collected 3 days after intra-dermal injection with tuberculin. Before MV infection classical delayed-type hypersensitivity responses were observed, associated with diffuse swelling and redness (indicated by arrows), after MV only a small localized papule was observed (indicated by arrow). H&E and CD3 staining of the corresponding skin tissues showed infiltration of T-lymphocytes in the dermis of the pre-infection Mantoux response, which was absent after MV infection. Representative images from 4 animals are shown.

*In vitro* and *in vivo* recall T-lymphocyte responses to tuberculin were measured in Bacille Calmette-Guérin (BCG)-vaccinated macaques, prior to and 11 or 13 d.p.i. IFN-γ production of PBMC in response to purified-protein derivative (PPD) stimulation was reduced after MV infection. Notably, an MV-specific IFN-γ response was detected in PBMC collected from macaques sacrificed 13 d.p.i. (Figure 4A). The *in vivo* recall response was determined by Mantoux testing. Before MV infection a characteristic delayed-type hypersensitivity response developed on the site of intra-dermal tuberculin injection, characterized by a well-delineated soft swelling of the cutus, corresponding with an influx of CD3^+^ T-lymphocytes (Figure 4B). In line with classical observations [4], [5], Mantoux responses were suppressed after MV infection, with a much smaller and harder swelling in the cutus, potentially corresponding with epidermal repair, skin-infiltrating CD3^+^ T-lymphocytes were absent (Figure 4B). Interestingly, we also observed macroscopically detectable EGFP expression in the skin at the sites where the animals had been vaccinated intra-cutaneously with BCG three months prior to MV infection (Figure S3), suggesting infection of tissue-resident memory lymphocytes in the skin.

### MV infection causes transient leukopenia followed by massive lymphocyte expansion

**Figure 5:**
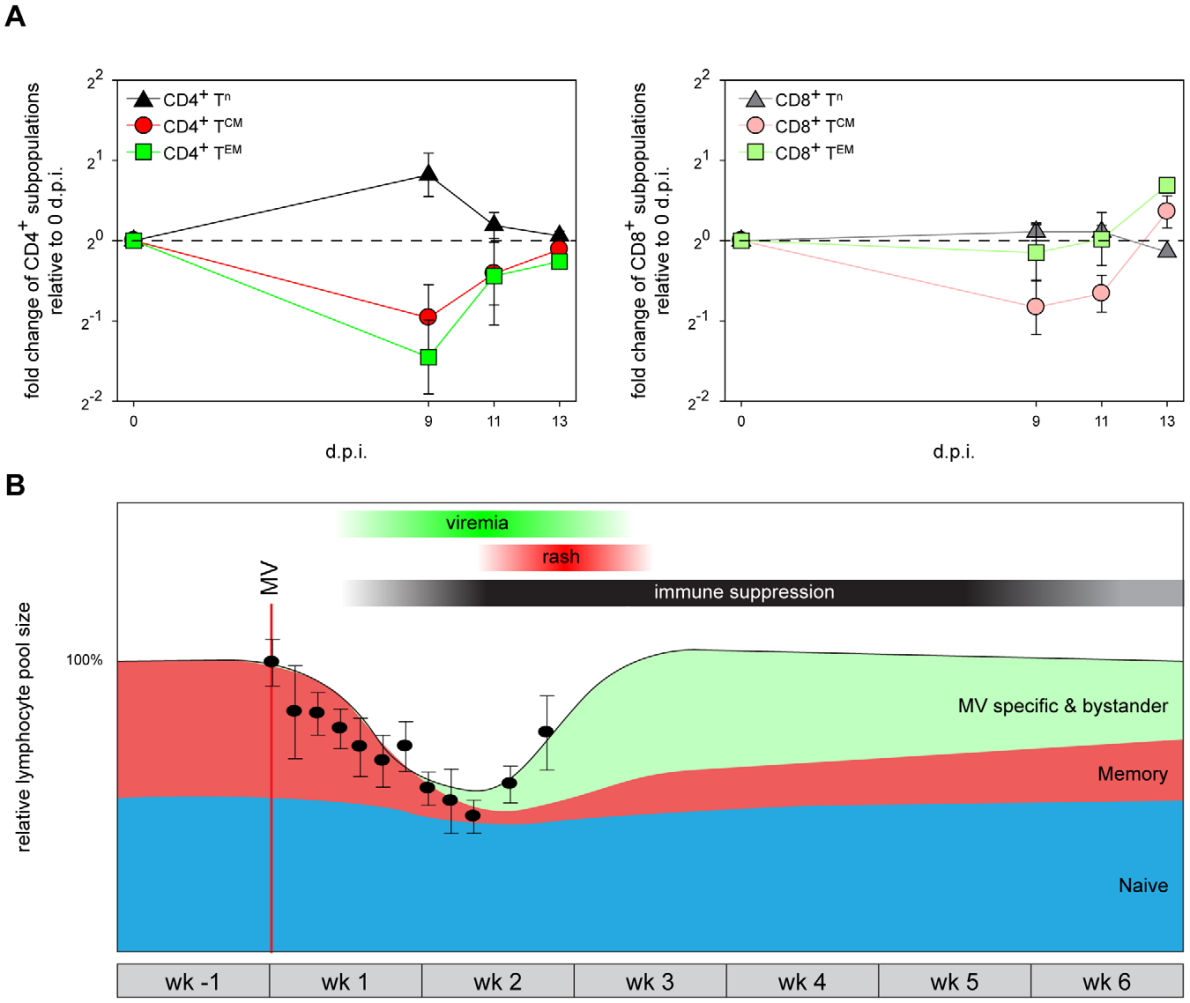
A model for measles immune suppression. (A) Relative population sizes of CD4^+^ or CD8^+^ T^n^, T^CM^ and T^EM^ in PBMC at different d.p.i., expressed as fold changes relative to 0 d.p.i. Means ± SEM of 9 animals are shown. (B). A model describing the changes in the relative size of pre-existing naive lymphocytes (blue), pre-existing memory lymphocytes (red, sum of T^M^ and follicular B-lymphocytes) and newly induced MV-specific (and bystander) lymphocytes (green) before, during and after measles. The relative WBC counts obtained from the macaques included in this study have been overlaid (black circles, means ± SEM). Thirty-four animals were included to obtain the WBC count graph. The red line indicates the time-point of MV-infection, bars above the graph indicate the approximate period of MV viremia (green), rash (red) and immune suppression (black).

Analysis of macaque white blood cell (WBC) counts during the acute infection demonstrated a profound but transient leukopenia (Figure 5B, circles), which coincided with the peak of viremia. There was a relative decrease in size of the CD45RA^−^ CD4^+^ and CD8^+^ T^CM^ and T^EM^ populations between 0 and 9 d.p.i. (Figure 5A), suggesting that leukopenia was related to depletion of MV-infected cells. WBC counts rapidly returned to pre-infection levels between 9 and 15 d.p.i. (Figure 5B), paralleled by a restoration of the relative CD45RA^−^ T^CM^ and T^EM^ population sizes (Figure 5A). Lymph nodes were enlarged in all animals euthanized between 11 and 15 d.p.i., which was paralleled by the clearance of EGFP^+^ lymphocytes from lymphoid tissues. During this period large numbers of Ki67^+^ proliferating cells repopulated the B-cell follicles in the germinal centers of lymphoid tissues (Figure 3; 13 and 15 d.p.i.). Numbers of apoptotic lymphocytes, as detected by staining for cleaved caspase 3 (CC3), remained low at all time-points (Figure S4). Infiltration of CD3^+^ T-lymphocytes into the B-cell follicles suggests that MV-infected cells were cleared by cytotoxic T-lymphocyte-mediated killing, rather than undergoing apoptosis [35].

### A unified model for measles immune suppression

Based on the observations described above we propose a model for the events leading to measles immune suppression. Infection and subsequent immune-mediated clearance of CD150^+^ lymphocytes results in specific depletion of memory T-lymphocytes and follicular B-lymphocytes, whilst the naive T-lymphocyte population remains relatively unaffected (Figure 5B, populations shown in red and blue, respectively). However, this leaves the question how such a short duration of measles lymphopenia can be reconciled with the long-lasting immune suppression. We hypothesize that the lymphocyte depletion is masked by the massive expansion of MV-specific and bystander lymphocytes (Figure 5B, population shown in green), which has also been observed in humans by a massive expansion of CD8^+^ T-lymphocytes [36] and by demonstrating skewing of the T-cell receptor repertoire after measles [37]. As a consequence, the qualitative composition of the lymphocyte population immediately after recovery from lymphopenia is dramatically different from that before MV infection, as pre-existing memory lymphocytes have been depleted and replaced by MV-specific and bystander lymphocytes. The net result is a temporary immunological amnesia, and restoration of immunological memory may take several weeks. Thus this model explains why measles-associated immune suppression extends well beyond the transient lymphopenia.

## Discussion

Based on our observations, we conclude that measles immune suppression can, at least in part, be explained by massive infection and subsequent immune-mediated clearance of CD150^+^ memory T-lymphocytes and follicular B-lymphocytes. Depletion of T- and B-lymphocytes has also been described in the BALT of measles patients [38]. We show that MV preferentially infects CD45RA^−^ T^CM^ and T^EM^, which during secondary immune responses are the primary source of T-lymphocyte expansion or generation of effector T-lymphocytes, respectively [39]. Infection and subsequent immune-mediated depletion of memory T-lymphocyte subsets fits with the first description of measles-induced immune suppression, namely the disappearance of Mantoux responses in measles patients [4].

Several hypotheses for the underlying mechanism of measles immune suppression have been described previously. However, none of these adequately explain the “measles paradox”: the disease is associated with immune suppression but also with induction of strong MV-specific immune responses. Our observations not only explain the measles paradox, but also shed light on many of the *in vivo* and *in vitro* correlates of measles immune suppression described in literature [40]–[42]. Unresponsiveness of PBMC to mitogens and altered cytokine profiles during and after the acute phase of measles have been demonstrated *in vitro* in several studies [10], [11]. These observations are not disputed, but we consider it difficult to extrapolate unresponsiveness of PBMC to mitogens *in vitro* to immune suppression *in vivo*. In parallel with the clearance of MV-infected cells we observed high numbers of proliferating Ki67^+^ cells in B-cell follicles. This fits well with classical observations of immune activation following measles [16], suggesting that lymphoproliferation is not impaired *in vivo* during the convalescent phase. The observed *in vitro* suppression of mitogen-induced lymphoproliferation could also be explained by the altered qualitative composition of lymphocyte populations in convalescent measles patients, compared to healthy controls. Our model and previously published observations [16] show that a large proportion of the lymphocytes that circulate during the first weeks after measles infection have been recently activated *in vivo*, potentially making these cells less susceptible to re-stimulation *in vitro*.

We do not exclude that a functional impairment of lymphocytes or DC contributes to immune suppression and thus may augment the extent of immune suppression. Actually, it is likely that there are many factors that contribute to immune suppression *in vivo*. To date, direct evidence of DC infection by MV in humans has not been obtained. However, it has been demonstrated *in vitro* that MV is capable of infecting mature DC and Langerhans cells (LC) [13], [14], [43]–[47]. *In vivo* in experimentally MV-infected macaques there was strong evidence for infection of DC in the skin and secondary lymphoid tissues [27]. The role of DC in immune suppression has not been extensively studied *in vivo*, but it is possible that they play a role either by directly being targeted and depleted by MV or indirectly by interaction with and silencing of T-lymphocytes. Furthermore, the capacity to function as antigen presenting cells might be affected [40], [48].

Our study covers a time period of two weeks after MV infection, and as such does not provide experimental proof of what happens during resolution of measles immune suppression. The strength of our model using recombinant EGFP-expressing MV mainly lies in the sensitive detection of MV-infected cells, which is limited to the first two weeks after MV infection. Clearly, the memory lymphocyte populations specific for previously encountered pathogens are not completely depleted, and are largely restored during the weeks to months after measles. For instance, previously positive Mantoux responses disappear after onset of rash [4], [5], but eventually reappear. We have indicated this in our model (Figure 5B) by showing a gradual increase of the memory lymphocyte population after clearance of MV. Although we cannot fully explain the drivers of the resolution of immune suppression, it is possible that expansion of non-depleted T^CM^ upon renewed antigen encounter may play an important role. However, homeostatic restoration by the immune system itself could be an alternative explanation.

Some of the individual observations described here have been reported earlier in relationship to animal morbillivirus-related immune suppression [49]–[51]. The novelty of our model lies in the immune-mediated lymphodepletion being masked by the massive expansion of MV-specific and bystander lymphocytes. Although effective MV-specific CD8^+^ T-lymphocyte responses as well as immune activation and lymph node enlargement have been described earlier [16], [36], [37], [51], they have not been associated with immune suppression in this way.

The most important consequence of our model is that the qualitative composition of lymphocyte populations changes dramatically upon MV infection. Although lymphocyte numbers in peripheral blood and lymphoid organs appear normal, depletion of pre-existing specific T- and B-lymphocytes subpopulations provides a direct explanation for the suppression of recall responses to other pathogens during and after measles. This allows such pathogens to cause severe disease and in the developing world leads to the high level of MV-associated mortality. In addition, MV efficiently replicates in B-lymphocytes, resulting in follicular exhaustion and disorganization of the germinal centers, which are essential in actively ongoing humoral immune responses.

We observed comparable levels of MV-infected cells in T^CM^, T^EM^ and B^n^, and in parallel observed lymphocyte depletion and disorganization in B-cell follicles during the acute phase of MV infection (Figure 3, 9–11 d.p.i.). However, we have also shown that proliferating cells can be detected in lymphoid tissues as early as 11 d.p.i., after which time the follicle structure is being restored. This matches the kinetics of antibody responses in the macaque model, in that MV-specific IgM and IgG responses are first detected around 11 d.p.i. and peak at 17 (IgM) and 24 (IgG) d.p.i. [27], [52]. These kinetics fit well with our conclusions from the data and map well onto the immune suppression model.

It has been described that MV infection can result in transient remissions of certain autoimmune diseases [53]–[55]. Our observations suggest that this can be explained by direct MV infection of CD150^+^ lymphocytes, followed by immune-mediated depletion. Similarly, this mechanism could also explain reductions in HIV-1 loads during acute measles [56], [57]: MV infection of memory CD4^+^ T-lymphocytes could result in depletion of HIV-1-infected cells. In certain auto-immune diseases, and in animal studies in which lymphocyte populations were experimentally depleted, commensal or opportunistic infectious agents that would normally be controlled by the immune system have been shown to cause severe disease [58]. The high incidence of respiratory and gastro-intestinal complications following measles [1]–[3] may therefore be directly related to the observed high percentages of MV-infection and subsequent lymphocyte depletion in the adenoids, tonsils and gut-associated lymphoid tissue, which form a first line of defense against inhaled or ingested pathogens. We conclude that MV infection wipes immunological memory, resulting in increased susceptibility to commensal or opportunistic infections.

## Materials and Methods

### Ethics statement

Animal experiments were conducted in compliance with European guidelines (EU directive on animal testing 86/609/EEC) and Dutch legislation (Experiments on Animals Act, 1997). The protocols were approved by the independent animal experimentation ethical review committee DCC in Driebergen, The Netherlands. Animal welfare was observed on daily basis, animal handling was performed under light anesthesia using ketamine and medetomidine. After handling atipamezole was administered to antagonize the effect of medetomidine. For experiments involving PBMC from human donors, written informed consent for research use was obtained by the Sanquin blood bank.

### Animal study design

PBMC and tissues were collected from cynomolgus (*Macaca fascicularis*) (n = 35) or rhesus (*Macaca mulatta*) (n = 5) macaques included in previously published studies [18], [27], [30] (n = 26) or from unpublished infection experiments with rMV^IC323^EGFP or rMV^KS^EGFP (Table S1 and Dataset S1) (n = 14). Macaques were infected by intra-tracheal inoculation or aerosol inhalation and euthanized at 2 (n = 3), 3 (n = 3), 4 (n = 3), 5 (n = 4), 7 (n = 9), 9 (n = 8), 11 (n = 6), 13 (n = 2) or 15 (n = 2) d.p.i. Although some of the experiments had been designed to address different research questions, the accumulated samples effectively covered all stages of MV infection in macaques.

### Mantoux tests

Four macaques received intra-dermal vaccinations with 4×0.1 ml of live BCG (NVI, Bilthoven, Netherlands). The animals received an intra-dermal Mantoux test with old tuberculin (0.1 ml, 25,000 IU/ml, Statens Serum Institut) [59] 3 months post-vaccination at 7 days pre-MV-infection, or 3 days prior to necropsy. Skin reactivity was assessed for three consecutive days. Skin samples from both Mantoux tests (pre- and post-MV infection) were collected into formalin.

### Tuberculin- and MV-specific T-lymphocyte responses

PBMC obtained 7 days pre-infection and during necropsy were thawed and plated into 96-wells round-bottom plates at 2×10^5^ cells per well. Cells were stimulated with either PPD (10 µg/ml), UV-inactivated MV (10 µg/ml) or live rMV^rEdt^EGFP [30] (5×10^4^ CCID_50_ in the presence of 10 µg/ml infection-enhancing lipopeptide PHCSK_4_ [60]) for 48 hours in triplicate. IFN-γ concentrations were measured in supernatants by ELISA (U-CyTech Biosciences).

### Leukopenia

After each blood collection, total WBC counts were obtained using an automated counter (Sysmex pocH-100iV). To address measles-induced leukopenia in these animals, mean WBC counts were determined on 0 (n = 31), 1 (n = 6), 2 (n = 20), 3 (n = 17), 4 (n = 18), 5 (n = 11), 6 (n = 20), 7 (n = 10), 8 (n = 6), 9 (n = 12), 11 (n = 8) and 13 (n = 2) d.p.i. Different animals were included at each time-point.

### Necropsy

Animals were euthanized by exsanguination under ketamine anesthesia. For the purpose of detecting EGFP fluorescence, a lamp was custom-made containing six 5-volt LEDs (Luxeon Lumileds, lambertian, cyan, peak emission 490–495 nm) mounted with D480/40 bandpass filters (Chroma) in a frame that allowed decontamination with 70% (v/v) alcohol or fumigation with formaldehyde. Emitted fluorescence was visualized through the amber cover of a UV transilluminator normally used for screening DNA gels. Photographs were made using a Nikon D80 digital SLR camera. Lymphoid tissues were collected in buffered formalin for immunohistochemistry or PBS for preparation of single cell suspensions, which were used directly for flow cytometry.

### Flow cytometry

T-lymphocytes were subdivided into T^n^, T^CM^ and T^EM^ populations (Figure S1) by staining with CD3^PerCP^ (BD Biosciences, clone SP34-2), CD4^V450^ (BD Biosciences, clone L200), CD8^AmCyan^ (BD Biosciences, clone SK1), CD45RA^PE-Cy7^ (BD Biosciences, clone L48) and CCR7^APC^ (R&D Systems, clone 150503). The APC signal was enhanced using an APC-FASER Kit (Miltenyi Biotec). B-lymphocytes were subdivided into B^n^ and B^M^ populations (Figure S1) by staining with CD20^PE-Cy7^ (BD Biosciences, clone L27), HLA-DR^Pacific Blue^ (Biolegend, clone L243), CD27^APC^ (eBioscience, clone O323) and a combination of IgD^Biotin^ (Southern Biotech, goat polyclonal) and streptavidin^PerCP^ (BD Biosciences). CD150 expression was determined by staining with CD150^FITC^ (AbD Serotec, clone A12). The infection percentages within the populations were determined by detection of EGFP. All flow cytometry was performed on a FACS Canto II (BD Biosciences).

### Histological and immunohistochemical analysis

H&E staining was performed to evaluate histological changes. Immunohistochemical staining was performed using a fully automated BondMax immunostainer with a polymer-based peroxidase detection system. MV-infected cells were detected using a polyclonal rabbit antibody to EGFP (Invitrogen). Similar stainings were performed with the following monoclonal antibodies: T-lymphocyte marker CD3 (DAKO, clone F7.2.38), B-lymphocyte marker CD20 (DAKO, clone L26), proliferation marker Ki67 (DAKO, clone MIB1) and apoptosis marker cleaved caspase 3 (Cell Signaling, clone 5A1E). Glass slides were scanned with a 40X/0.75 Olympus UPlan FLN objective on an Aperio Scanscope CS-O SS5200 equipped with Spectrum Plus. An Aperio Positive Pixel Count Algorithm was applies to quantify and therefore standardize the intensity of stains present to produce optimal discrimination between immunoperoxidase diaminobenzidine (DAB) tetrahydrochloride reactions and hematoxylin stained nuclei. Scanners are kept at ambient temperature in a temperature-controlled area to eliminate loss of performance due to overheating.

### Susceptibility of lymphocyte subsets to infection

PBMC from healthy human or macaque donors were sorted into pure CD4^+^ or CD8^+^ naive (CD45RA^+^) and memory (CD45RA^−^) T-lymphocyte populations on basis of CD45RA expression. Unsorted or sorted PBMC from humans or macaques were infected by co-culture with low numbers (1∶1000) of autologous MV-infected B-LCL or BAL cells (infected with cell-free rMV^KS^EGFP at an MOI of 1 for 48 hours), respectively. After two days of co-culture infection percentages in the different subsets were determined by flow cytometry.

### Statistical analysis

Differences between percentages infected cells or CD150 expression were tested by the non-parametric Wilcoxon rank test using SPSS software.

## Supporting Information

Dataset S1 **Virus isolation data from BAL cells and PBMC of macaques included in this study.** Small-volume EDTA blood samples were collected in Vacuette tubes. PBMC were isolated by density gradient centrifugation, resuspended in culture medium and used for virus isolation. BAL was performed by intra-tracheal infusion of 10 ml PBS through a flexible catheter and immediate recovery. Recovered BAL cells were resuspended in culture medium, counted, and used directly for virus isolation. Isolation of MV was performed on Vero-CD150 cells using an infectious center assay. Virus isolations were monitored by UV microscopy for EGFP fluorescence for 3 to 6 days. Results are expressed as the number of MV-infected cells per 10^6^ total cells. In different animal experiments, BAL and PBMC samples were collected on different time points after MV infection. Available data per time point were used to calculate the median virus loads, which are shown as green and red area curves for BAL cells and PBMC, respectively. Median virus loads determined in BAL cells or PBMC peaked 9 d.p.i, and virus loads were higher in BAL cells than in PBMC. In addition to the median virus loads, each plot shows the virus isolation data obtained for one individual animal; animals are numbered according to the first column of Table S1. The green circles and red triangles show the virus loads in BAL cells and PBMC, respectively. These data are included to provide background information on the level of individual variation between outbred non-SPF animals of different species and infected with different virus strains.
(DOC)

Figure S1 **Gating strategy used to distinguish between different T- and B-lymphocyte subsets.** As a first step, the lymphocyte population was gated on basis of forward and side scatter (FSC and SSC, respectively). (A) T-lymphocyte subpopulations were subsequently detected on basis of expression of CD3 and CD4 or CD8, and identified as naive T-lymphocytes (T^n^, CD45RA^+^), central memory T-lymphocytes (T^CM^, CD45RA^−^CCR7^+^) or effector memory T-lymphocytes (T^EM^, CD45RA^−^CCR7^−^). The monoclonal antibodies used (see materials and methods for clone numbers) cross-react with rhesus and cynomolgus macaque antigens (nhpreagentsbidmc.harvard.edu/), and identified similar lymphocyte populations as previously described for human T-lymphocytes. (B) B-lymphocytes were detected on basis of expression of CD20 and HLA-DR, and identified as naive B-lymphocytes (B^n^, CD27^−^IgD^+^) or memory B-lymphocytes (B^M^, CD27^+^IgD^−^) as previously described. EGFP^+^ cells were gated to determine the level of MV infection within each lymphocyte subset. In some cases cells expressing high levels of EGFP were found to run “off-scale” (see upper right plot), but these events could be included in the analysis of the percentage EGFP^+^ cells.
(TIF)

Figure S2 **Dual immunofluorescence staining of lymphoid tissues obtained from macaques euthanized 7 d.p.i.** (A–C) Large numbers of multinucleated syncytia were observed in the B-cell follicles and were stained for EGFP (green) as a marker of MV infection. Double stains were performed with a B-lymphocyte marker (CD20, red, A), a T-lymphocyte marker (CD3, red, B) or a macrophage/DC marker (CD11c, red, C) and DAPI was used to counterstain the nuclei (blue). Left panels only show the red and blue channels, right panels show the combined red, blue and green channels. Multi-nucleated giant cells were mainly of B-lymphocyte origin (panel A), and the infection was associated with significant cytopathic effects in lymphoid tissues.
(TIF)

Figure S3 **Macroscopic detection of EGFP at BCG intra-dermal injection sites (indicated by arrows).** (A) Corresponding normal and (B) fluorescent macroscopic photographs of BGC-injection sites. To study MV infection of pre-existing specific memory lymphocytes, four macaques were vaccinated intra-dermally with BCG three months prior to MV infection. Vaccination with this live-attenuated bacterial vaccine resulted in macroscopically detectable local inflammatory responses, which remained detectable for several weeks post-vaccination. After MV infection, EGFP fluorescence was observed macroscopically in the skin at the BCG vaccination sites 9 d.p.i. This was due to the presence of EGFP^+^ lymphocytes (not shown), suggesting that the virus targeted the BCG-specific tissue-resident memory T-lymphocytes. We have previously described the presence of MV-infected aggregates of lymphoid cells in the skin of macaques [27], but were unable to determine the answer to the “chicken or egg” question: were these cells present in the skin before MV infection and subsequently targeted by the virus, or did they infiltrate into the skin after MV infection? The observed infection of lymphocytes in the skin at the location where the animals had been intra-dermally immunized with BCG three months earlier strongly suggests that these lymphocytes were present in the skin and subsequently targeted by the virus.
(TIF)

Figure S4 **Immunohistochemical staining of lymphoid tissues obtained from macaques euthanized between 5 and 15 d.p.i.** Apoptotic cells were visualized indirectly using monoclonal antibody against CC3 and DAB-detection. The same animals and lymphoid tissues used in Figure 4 were analyzed and panels shown are representative for the tissues that have been examined. The same B-cell follicle is shown at ×10 and ×20 magnification. The bar below the photomicrographs of the CC3 staining indicates the relative level of apoptosis, as was done for MV-infection, B-cell depletion, T-cell depletion and proliferation in Figure 4. Note that there was no change in the numbers of apoptotic cells within B-cell follicles during the time-course of MV infection. The green and red bars at the bottom indicate viremia and rash, and correspond to the bars in Figure 5B. Examples of CC3-positive cells are indicated by arrows in the lower left panel at 5 d.p.i. These stainings show that the depletion of B-cell follicles is not caused by apoptosis of infected cells.
(TIF)

Table S1 **Overview of animals included in this study.** ^1^ Animal numbering for this manuscript, animals are ordered according to time point of necropsy; ^2^ Original animal number as described in previous manuscripts; ^3^ F = female, M = male, N = sex not recorded; ^4^ C = cynomolgus monkey (*Macaca fascicularis*), R = rhesus monkey (*Macaca mulatta*); ^5^ Age range, in months; ^6^ For description of viruses see references under ^10^; ^7^ Aerosol = inhalation of nebulized virus via a facemask, IT = intra-tracheal inoculation of the virus diluted in 5 ml phosphate buffered saline (PBS), for description of devices and methods see references under ^10^; ^8^ For aerosol administration higher doses were used than for IT administration, as it was estimated that approximately 1% of the nebulized dose would be inhaled into the lungs; ^9^ Time point of necropsy, shown in d.p.i.; ^10^ Reference to the study in which this animal was included.
(DOC)
