# Supplementary figures and images for: Measles Immune Suppression: Lessons from the Macaque Model
Source: PLoS Pathog. 2012 Aug 30;8(8):e1002885. doi: 10.1371/journal.ppat.1002885 (PMC3431343; doi:10.1371/journal.ppat.1002885)

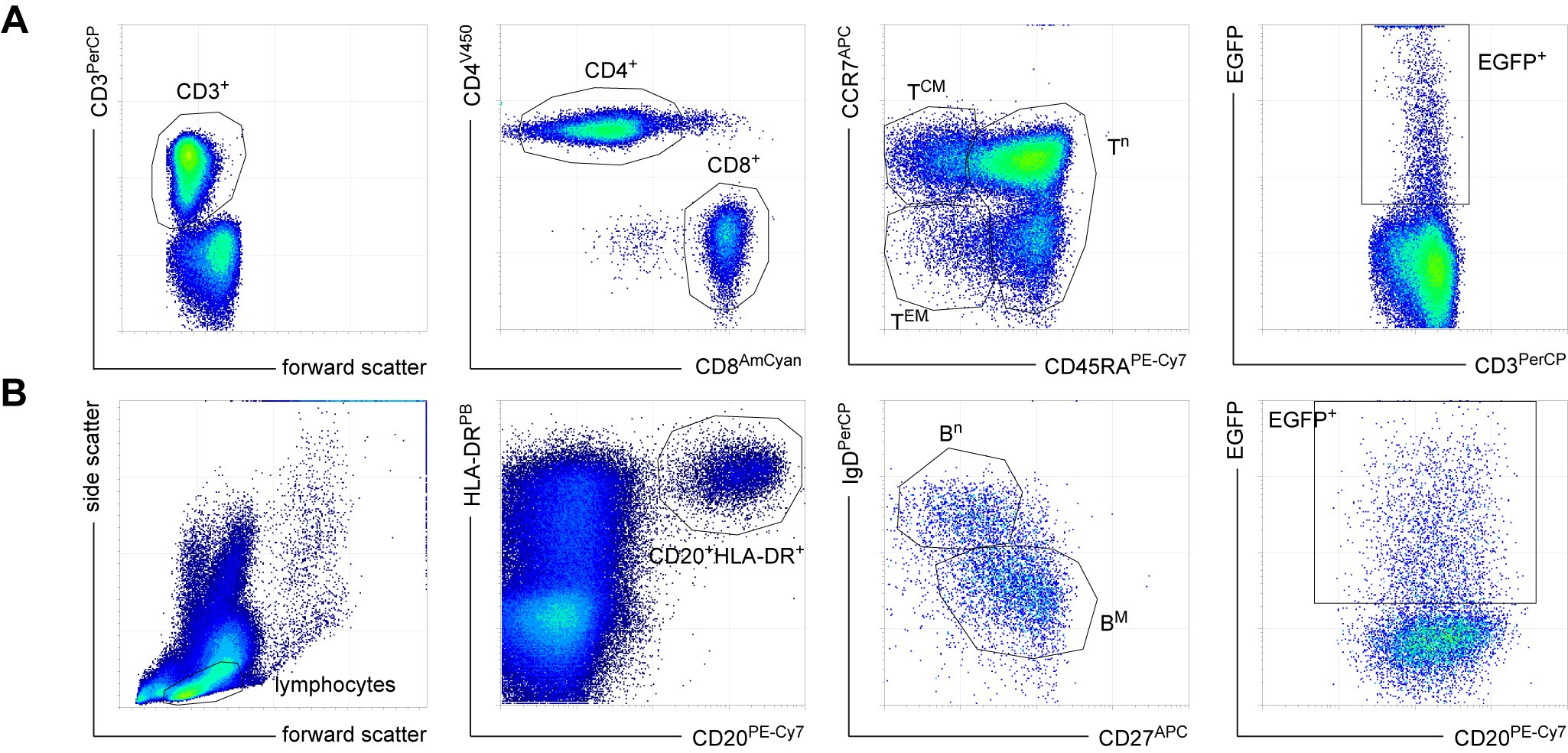

Supplement: Figure S1 — Gating strategy used to distinguish between different T- and B-lymphocyte subsets. As a first step, the lymphocyte population was gated on basis of forward and side scatter (FSC and SSC, respectively). (A) T-lymphocyte subpopulations were subsequently detected on basis of expression of CD3 and CD4 or CD8, and identified as naive T-lymphocytes (Tn, CD45RA+), central memory T-lymphocytes (TCM, CD45RA−CCR7+) or effector memory T-lymphocytes (TEM, CD45RA−CCR7−). The monoclonal antibodies used (see materials and methods for clone numbers) cross-react with rhesus and cynomolgus macaque antigens (nhpreagentsbidmc.harvard.edu/), and identified similar lymphocyte populations as previously described for human T-lymphocytes. (B) B-lymphocytes were detected on basis of expression of CD20 and HLA-DR, and identified as naive B-lymphocytes (Bn, CD27−IgD+) or memory B-lymphocytes (BM, CD27+IgD−) as previously described. EGFP+ cells were gated to determine the level of MV infection within each lymphocyte subset. In some cases cells expressing high levels of EGFP were found to run “off-scale” (see upper right plot), but these events could be included in the analysis of the percentage EGFP+ cells. (TIF) [file ppat.1002885.s002.tif]

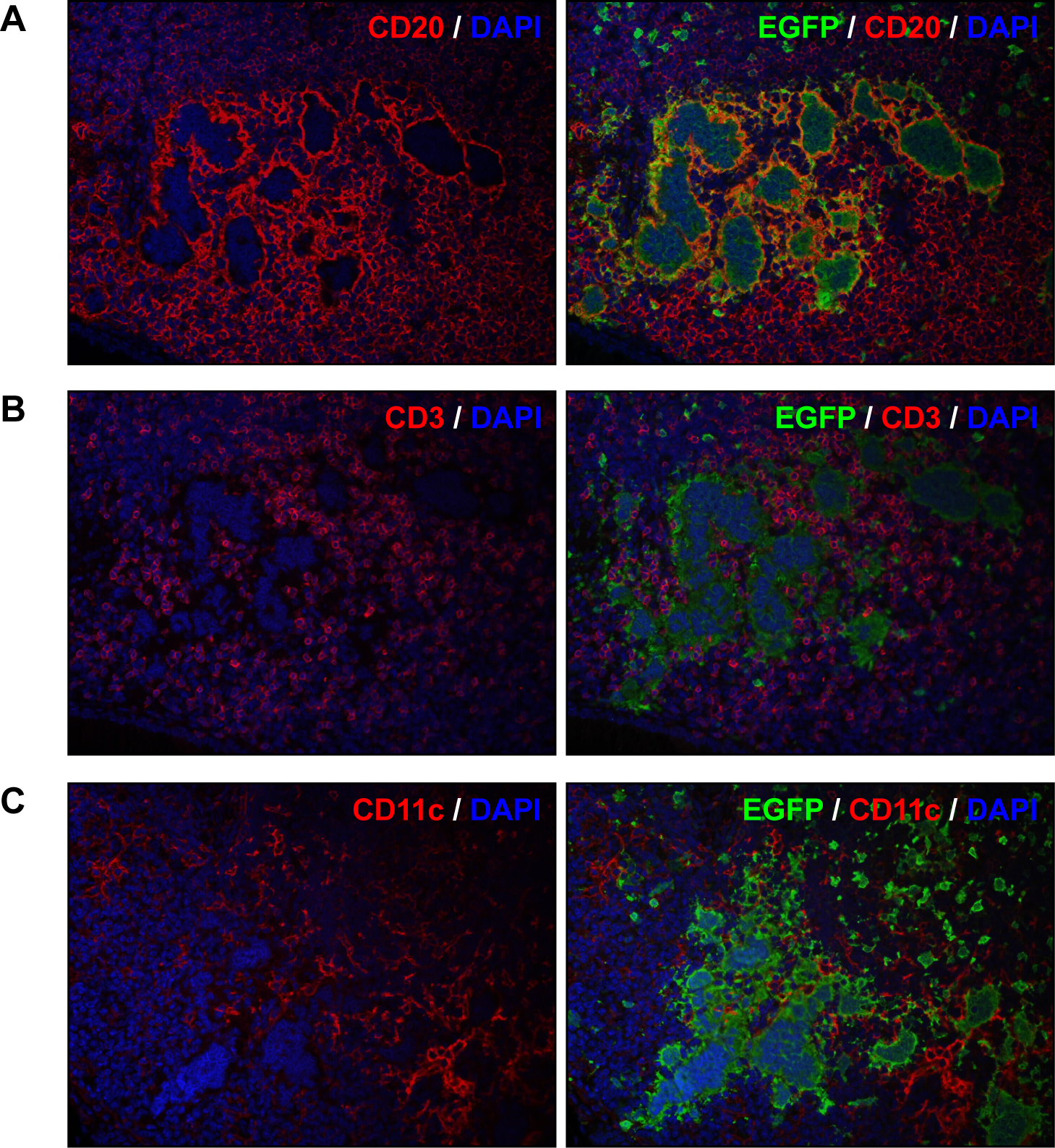

Supplement: Figure S2 — Dual immunofluorescence staining of lymphoid tissues obtained from macaques euthanized 7 d.p.i. (A–C) Large numbers of multinucleated syncytia were observed in the B-cell follicles and were stained for EGFP (green) as a marker of MV infection. Double stains were performed with a B-lymphocyte marker (CD20, red, A), a T-lymphocyte marker (CD3, red, B) or a macrophage/DC marker (CD11c, red, C) and DAPI was used to counterstain the nuclei (blue). Left panels only show the red and blue channels, right panels show the combined red, blue and green channels. Multi-nucleated giant cells were mainly of B-lymphocyte origin (panel A), and the infection was associated with significant cytopathic effects in lymphoid tissues. (TIF) [file ppat.1002885.s003.tif]

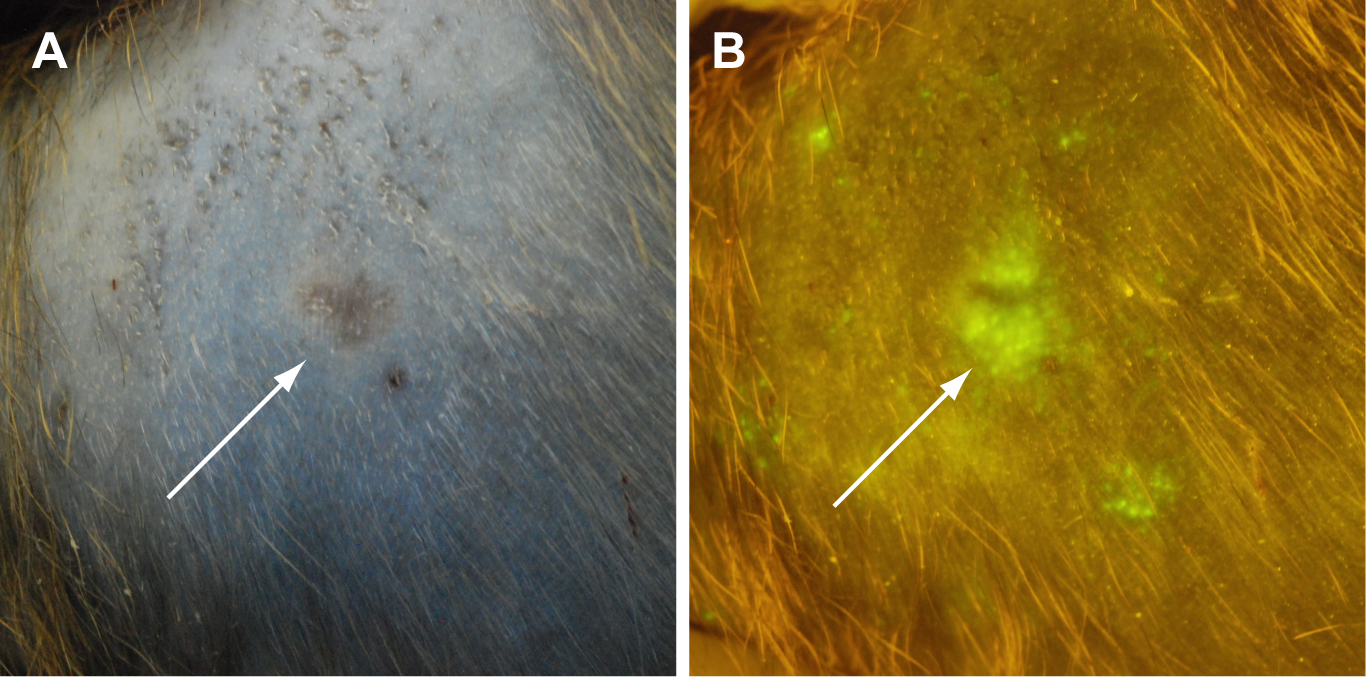

Supplement: Figure S3 — Macroscopic detection of EGFP at BCG intra-dermal injection sites (indicated by arrows). (A) Corresponding normal and (B) fluorescent macroscopic photographs of BGC-injection sites. To study MV infection of pre-existing specific memory lymphocytes, four macaques were vaccinated intra-dermally with BCG three months prior to MV infection. Vaccination with this live-attenuated bacterial vaccine resulted in macroscopically detectable local inflammatory responses, which remained detectable for several weeks post-vaccination. After MV infection, EGFP fluorescence was observed macroscopically in the skin at the BCG vaccination sites 9 d.p.i. This was due to the presence of EGFP+ lymphocytes (not shown), suggesting that the virus targeted the BCG-specific tissue-resident memory T-lymphocytes. We have previously described the presence of MV-infected aggregates of lymphoid cells in the skin of macaques [27], but were unable to determine the answer to the “chicken or egg” question: were these cells present in the skin before MV infection and subsequently targeted by the virus, or did they infiltrate into the skin after MV infection? The observed infection of lymphocytes in the skin at the location where the animals had been intra-dermally immunized with BCG three months earlier strongly suggests that these lymphocytes were present in the skin and subsequently targeted by the virus. (TIF) [file ppat.1002885.s004.tif]

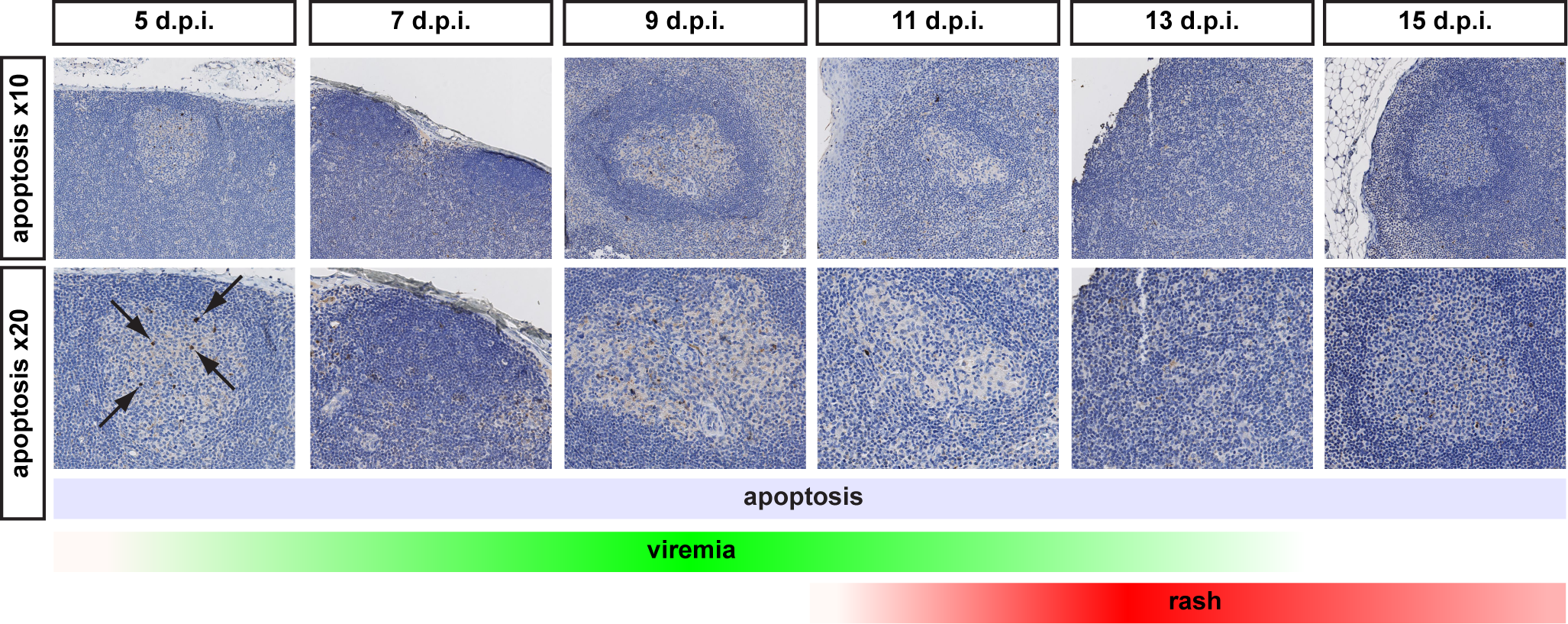

Supplement: Figure S4 — Immunohistochemical staining of lymphoid tissues obtained from macaques euthanized between 5 and 15 d.p.i. Apoptotic cells were visualized indirectly using monoclonal antibody against CC3 and DAB-detection. The same animals and lymphoid tissues used in Figure 4 were analyzed and panels shown are representative for the tissues that have been examined. The same B-cell follicle is shown at ×10 and ×20 magnification. The bar below the photomicrographs of the CC3 staining indicates the relative level of apoptosis, as was done for MV-infection, B-cell depletion, T-cell depletion and proliferation in Figure 4. Note that there was no change in the numbers of apoptotic cells within B-cell follicles during the time-course of MV infection. The green and red bars at the bottom indicate viremia and rash, and correspond to the bars in Figure 5B. Examples of CC3-positive cells are indicated by arrows in the lower left panel at 5 d.p.i. These stainings show that the depletion of B-cell follicles is not caused by apoptosis of infected cells. (TIF) [file ppat.1002885.s005.tif]
