# Supplementary material for: Measles Immune Suppression: Lessons from the Macaque Model
Source: PLoS Pathog. 2012 Aug 30;8(8):e1002885. doi: 10.1371/journal.ppat.1002885 (PMC3431343; doi:10.1371/journal.ppat.1002885)
Supplement: Table S1 — Overview of animals included in this study. 1 Animal numbering for this manuscript, animals are ordered according to time point of necropsy; 2 Original animal number as described in previous manuscripts; 3 F = female, M = male, N = sex not recorded; 4 C = cynomolgus monkey (Macaca fascicularis), R = rhesus monkey (Macaca mulatta); 5 Age range, in months; 6 For description of viruses see references under 10; 7 Aerosol = inhalation of nebulized virus via a facemask, IT = intra-tracheal inoculation of the virus diluted in 5 ml phosphate buffered saline (PBS), for description of devices and methods see references under 10; 8 For aerosol administration higher doses were used than for IT administration, as it was estimated that approximately 1% of the nebulized dose would be inhaled into the lungs; 9 Time point of necropsy, shown in d.p.i.; 10 Reference to the study in which this animal was included. (DOC) [file ppat.1002885.s006.doc]

**Table S1: Overview of animals included in this study**

| **#1** | **ID^2^** | **Sex^3^** | **Sp^4^** | **Age^5^** | **Virus^6^** | **Route^7^** | **Dose^8^** | **Necropsy^9^** | **Ref^10^** |
| --- | --- | --- | --- | --- | --- | --- | --- | --- | --- |
| 1 | ET01 | F | C | 24-36 | rMV^KS^EGFP | aerosol | 10^6^ | 2 d.p.i. | Lemon *et al*., 2011 |
| 2 | ET02 | F | C | 24-36 | rMV^KS^EGFP | aerosol | 10^6^ | 2 d.p.i. | Lemon *et al*., 2011 |
| 3 | ET03 | M | C | 24-36 | rMV^KS^EGFP | aerosol | 10^6^ | 2 d.p.i. | Lemon *et al*., 2011 |
| 4 | ET04 | F | C | 24-36 | rMV^KS^EGFP | aerosol | 10^6^ | 3 d.p.i. | Lemon *et al*., 2011 |
| 5 | ET05 | M | C | 24-36 | rMV^KS^EGFP | aerosol | 10^6^ | 3 d.p.i. | Lemon *et al*., 2011 |
| 6 | ET06 | F | C | 24-36 | rMV^KS^EGFP | aerosol | 10^6^ | 3 d.p.i. | Lemon *et al*., 2011 |
| 7 | ET07 | F | C | 24-36 | rMV^KS^EGFP | aerosol | 10^6^ | 4 d.p.i. | Lemon *et al*., 2011 |
| 8 | ET08 | F | C | 24-36 | rMV^KS^EGFP | aerosol | 10^6^ | 4 d.p.i. | Lemon *et al*., 2011 |
| 9 | ET09 | F | C | 24-36 | rMV^KS^EGFP | aerosol | 10^6^ | 4 d.p.i. | Lemon *et al*., 2011 |
| 10 | ET10 | F | C | 24-36 | rMV^KS^EGFP | aerosol | 10^6^ | 5 d.p.i. | Lemon *et al*., 2011 |
| 11 | ET11 | F | C | 24-36 | rMV^KS^EGFP | aerosol | 10^6^ | 5 d.p.i. | Lemon *et al*., 2011 |
| 12 | ET12 | F | C | 24-36 | rMV^KS^EGFP | aerosol | 10^6^ | 5 d.p.i. | Lemon *et al*., 2011 |
| 13 | R4 | M | R | 24-48 | rMV^IC323^EGFP | aerosol | 10^6^ | 5 d.p.i. | this study |
| 14 | A1 | F | C | 30-48 | rMV^IC323^EGFP | IT | 10^4^ | 7 d.p.i. | De Vries *et al*., 2010a |
| 15 | A2 | F | C | 30-48 | rMV^IC323^EGFP | IT | 10^4^ | 7 d.p.i. | De Vries *et al*., 2010a |
| 16 | A3 | F | C | 30-48 | rMV^IC323^EGFP | IT | 10^4^ | 7 d.p.i. | De Vries *et al*., 2010a |
| 17 | A4 | M | C | 30-48 | rMV^IC323^EGFP | IT | 10^4^ | 7 d.p.i. | De Vries *et al*., 2010a |
| 18 | B1 | F | C | 30-48 | rMV^IC323^EGFP | aerosol | 10^6^ | 7 d.p.i. | De Vries *et al*., 2010a |
| 19 | B2 | F | C | 30-48 | rMV^IC323^EGFP | aerosol | 10^6^ | 7 d.p.i. | De Vries *et al*., 2010a |
| 20 | B3 | F | C | 30-48 | rMV^IC323^EGFP | aerosol | 10^6^ | 7 d.p.i. | De Vries *et al*., 2010a |
| 21 | B4 | F | C | 30-48 | rMV^IC323^EGFP | aerosol | 10^6^ | 7 d.p.i. | De Vries *et al*., 2010a |
| 22 | R5 | M | R | 24-48 | rMV^IC323^EGFP | aerosol | 10^6^ | 7 d.p.i. | this study |
| 23 | R1 | M | R | 24-48 | rMV^IC323^EGFP | IT | 10^4^ | 9 d.p.i. | De Swart *et al*., 2007 |
| 24 | C1 | N | C | 24-48 | rMV^IC323^EGFP | IT | 10^4^ | 9 d.p.i. | De Swart *et al*., 2007 |
| 25 | C3 | N | C | 24-48 | rMV^IC323^EGFP | IT | 10^4^ | 9 d.p.i. | De Swart *et al*., 2007 |
| 26 | R3 | N | R | 24-48 | rMV^IC323^EGFP | IT | 10^4^ | 9 d.p.i. | De Swart *et al*., 2007 |
| 27 | TIS01 | M | C | 24-36 | rMV^KS^EGFP | IT | 10^4^ | 9 d.p.i. | this study |
| 28 | TIS02 | F | C | 24-36 | rMV^KS^EGFP | IT | 10^4^ | 9 d.p.i. | this study |
| 29 | TIS03 | M | C | 24-36 | rMV^KS^EGFP | aerosol | 10^5^ | 9 d.p.i. | this study |
| 30 | TIS04 | M | C | 24-36 | rMV^KS^EGFP | aerosol | 10^5^ | 9 d.p.i. | this study |
| 31 | TIS05 | F | C | 24-36 | rMV^KS^EGFP | IT | 10^4^ | 11 d.p.i. | this study |
| 32 | TIS06 | F | C | 24-36 | rMV^KS^EGFP | IT | 10^4^ | 11 d.p.i. | this study |
| 33 | TIS07 | F | C | 24-36 | rMV^KS^EGFP | aerosol | 10^5^ | 11 d.p.i. | this study |
| 34 | TIS08 | M | C | 24-36 | rMV^KS^EGFP | aerosol | 10^5^ | 11 d.p.i. | this study |
| 35 | TIS09 | F | C | 24-36 | rMV^KS^EGFP | aerosol | 10^5^ | 11 d.p.i. | this study |
| 36 | C4 | M | C | 24-48 | rMV^IC323^EGFP | aerosol | 10^6^ | 11 d.p.i. | this study |
| 37 | TIS10 | F | C | 24-36 | rMV^KS^EGFP | IT | 10^4^ | 13 d.p.i. | this study |
| 38 | TIS11 | M | C | 24-36 | rMV^KS^EGFP | IT | 10^4^ | 13 d.p.i. | this study |
| 39 | R2 | M | R | 24-48 | rMV^IC323^EGFP | IT | 10^4^ | 15 d.p.i. | De Swart *et al*., 2007 |
| 40 | C2 | M | C | 24-48 | rMV^IC323^EGFP | IT | 10^4^ | 15 d.p.i. | De Swart *et al*., 2007 |
