# Supplementary material for: Measles Immune Suppression: Lessons from the Macaque Model
Source: PLoS Pathog. 2012 Aug 30;8(8):e1002885. doi: 10.1371/journal.ppat.1002885 (PMC3431343; doi:10.1371/journal.ppat.1002885)
Supplement: Supplementary file 3 [file ppat.1002885.s001.doc]

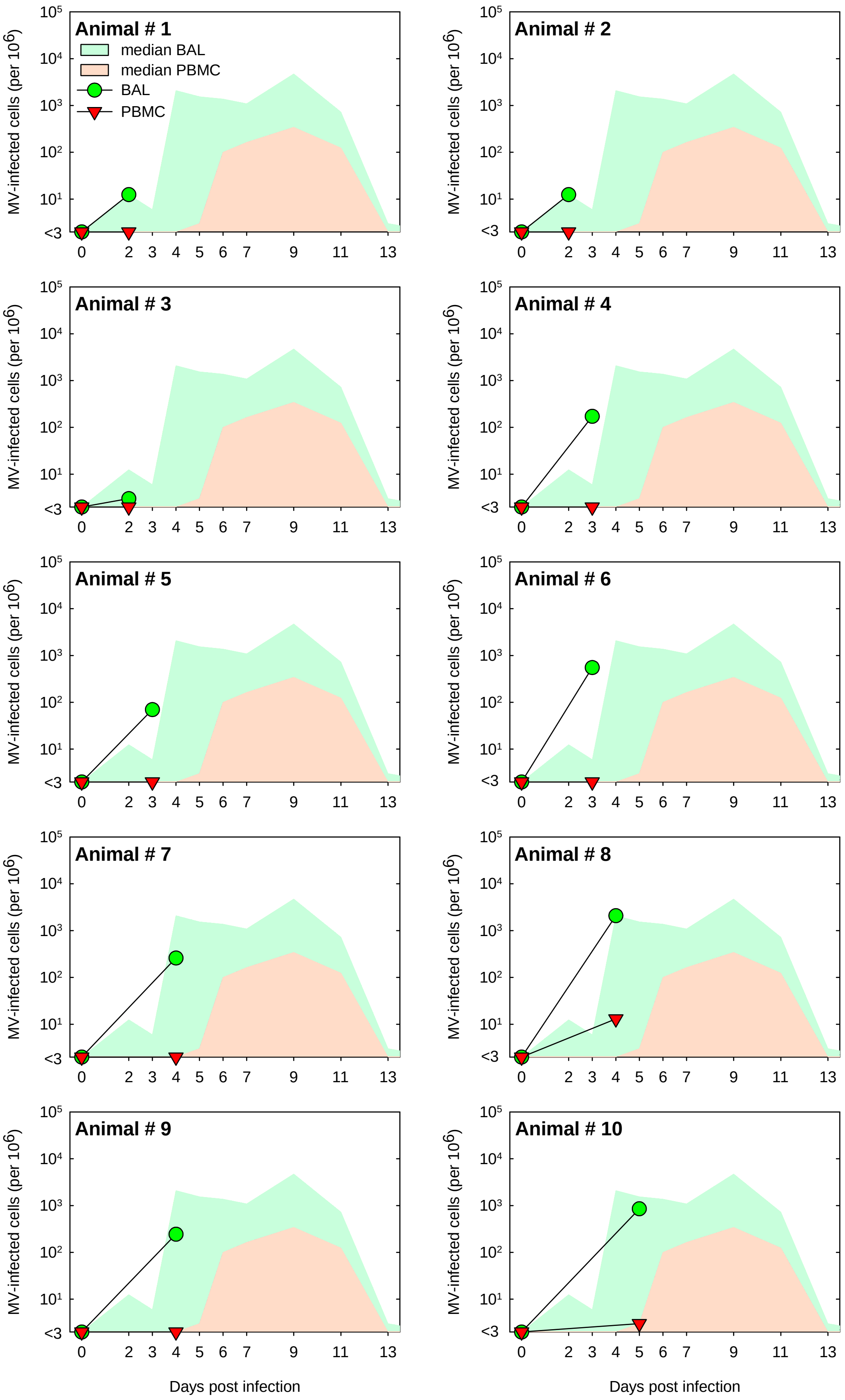
Animal # 1
median BAL
median PBMC
BAL
PBMC
Animal # 2
Animal # 3
Animal # 4
Animal # 5
Animal # 6
Animal # 7
Animal # 8
Animal # 9
Animal # 10
MV-infected cells (per 10^6)
Days post infection
10^5
10^4
10^3
10^2
10^1
<3
0 2 3 4 5 6 7 9 11 13

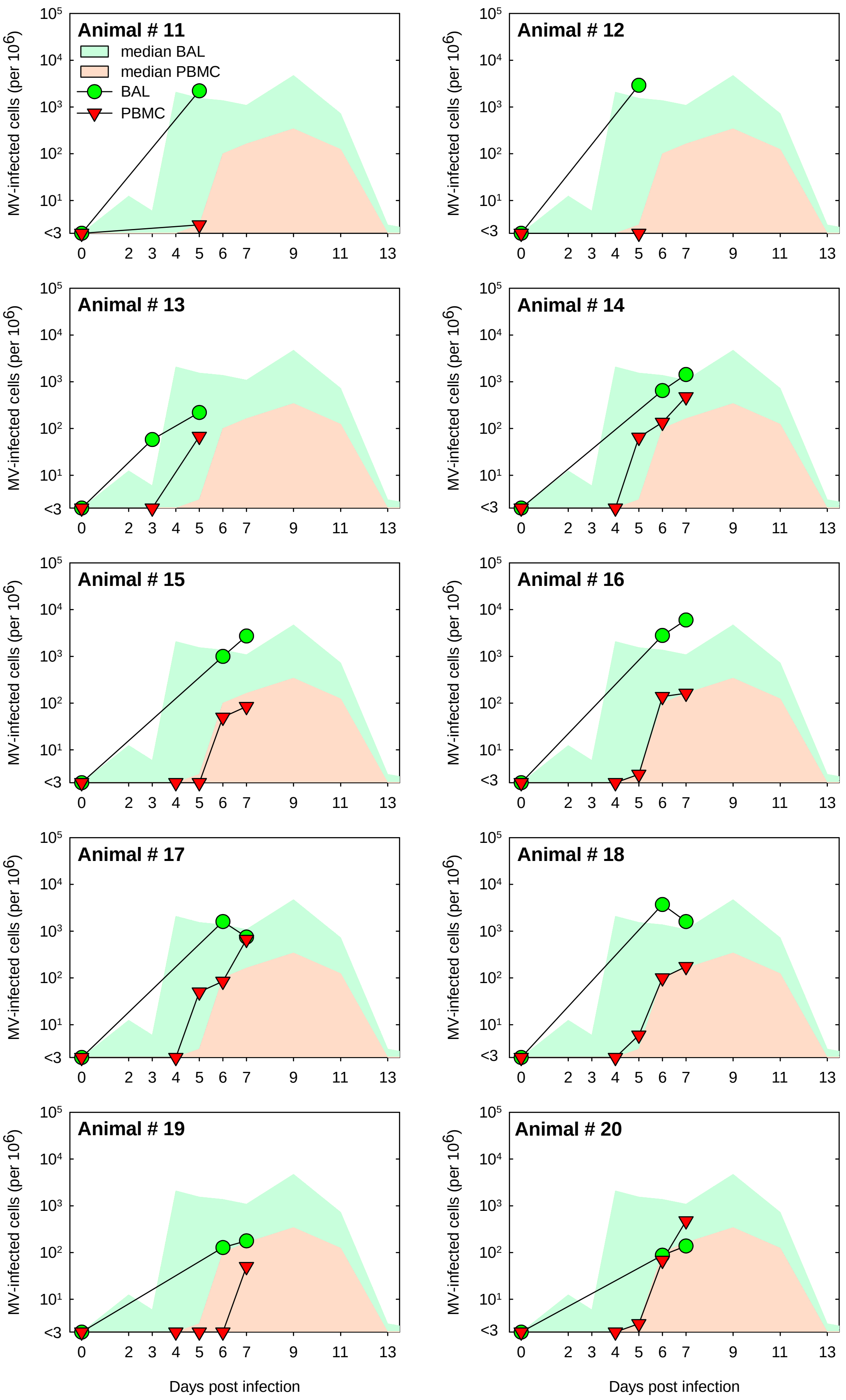
Animal # 11
median BAL
median PBMC
BAL
PBMC
Animal # 12
Animal # 13
Animal # 14
Animal # 15
Animal # 16
Animal # 17
Animal # 18
Animal # 19
Animal # 20
MV-infected cells (per 10^6)
10^5
10^4
10^3
10^2
10^1
<3
0 2 3 4 5 6 7 9 11 13
Days post infection

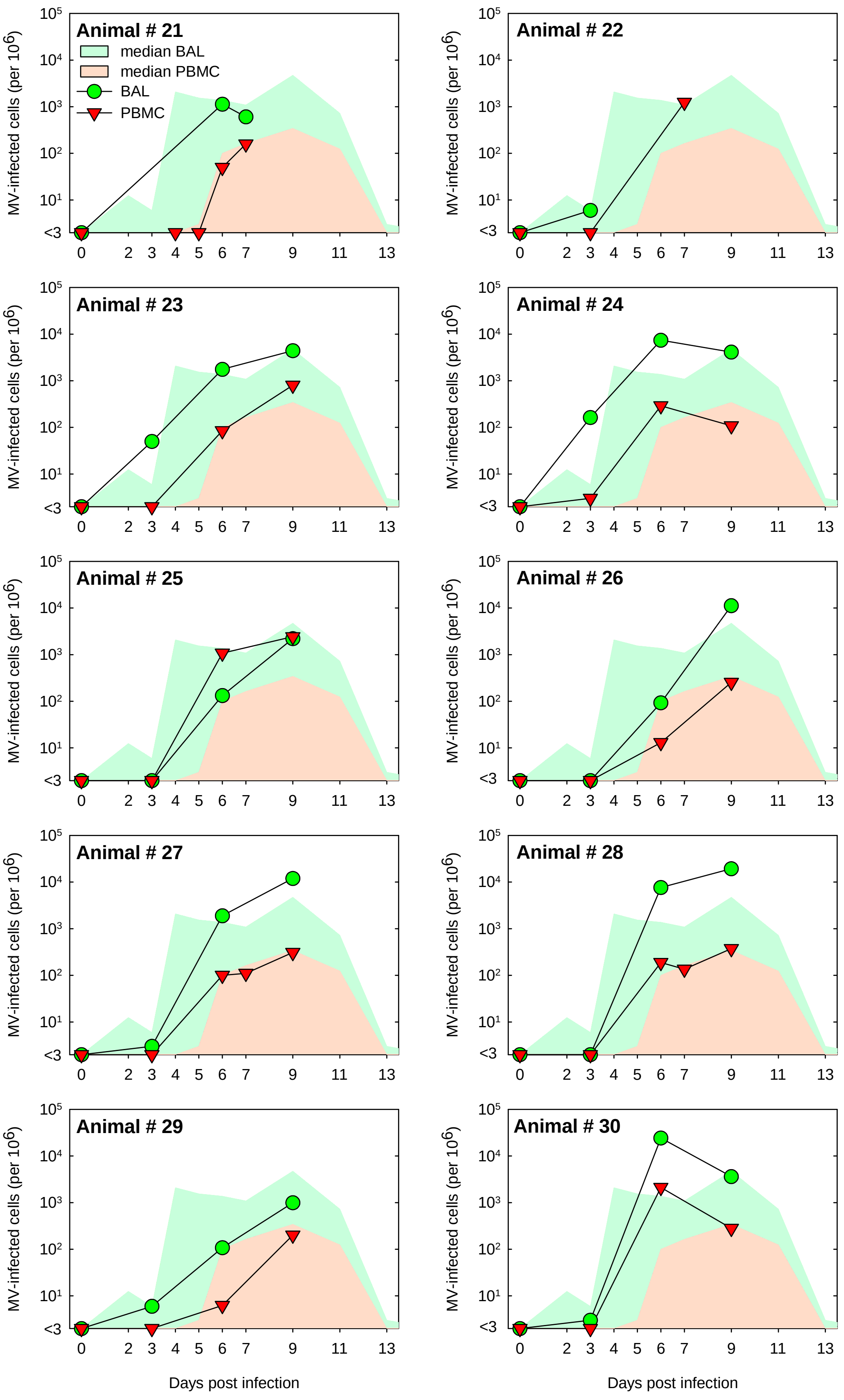
Animal # 21
median BAL
median PBMC
BAL
PBMC
Animal # 22
Animal # 23
Animal # 24
Animal # 25
Animal # 26
Animal # 27
Animal # 28
Animal # 29
Animal # 30
MV-infected cells (per 10^6)
<3
10^1
10^2
10^3
10^4
10^5
0
2
3
4
5
6
7
9
11
13
Days post infection

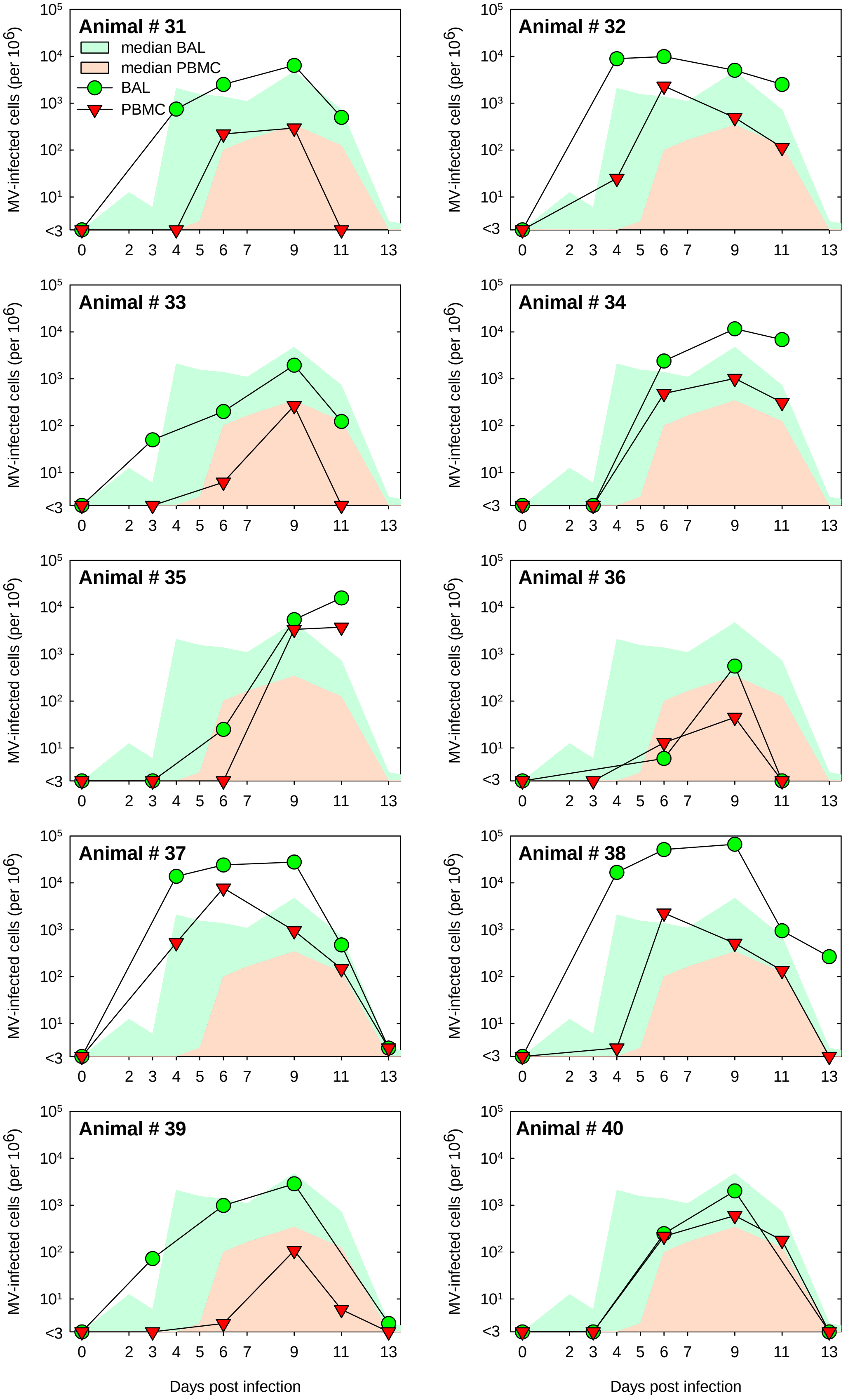
Animal # 31
median BAL
median PBMC
BAL
PBMC
Animal # 32
Animal # 33
Animal # 34
Animal # 35
Animal # 36
Animal # 37
Animal # 38
Animal # 39
Animal # 40
MV-infected cells (per 10^6)
Days post infection
